# Novel Use of *Ex Vivo* Uretero-Pyeloscopy in Autotransplantation: A Systematic Review and Case Report

**DOI:** 10.1089/cren.2015.0020

**Published:** 2015-12-01

**Authors:** Trevor Tnay, Sandra Elmer, Damien M. Bolton, Nathan Lawrentschuk

**Affiliations:** ^1^ Urology Unit, Department of Surgery, University of Melbourne, Melbourne, Australia.; ^2^ Austin Hospital, Olivia Newton-John Cancer Research Institute, Heidelberg, Australia.

## Abstract

***Background:*** Autotransplant has been practiced for decades but is regaining popularity in the nephron-sparing era. Initially for benign disease, autotransplantation has a select role in malignant processes that warrants new techniques and ideas to ensure patient safety. We review the use of *ex vivo* uretero-pyeloscopy and frozen section to ensure kidneys may be utilized in a patient with suspected malignancy.

***Case Presentation:*** A systematic review (PRISMA standard) of *ex vivo* uretreo–pyeloscopy was undertaken. We then present the case of a 37-year-old Caucasian female who was suspected of having ureteral obstructing malignancy; she had previous treatment of the bladder with bacillus Calmette–Guerin (BCG) for recurrent urothelial malignancy. The lesion biopsies and cytology were suspicious but inconclusive, indicating nephroureterectomy was a likely course of management.

***Results:*** On reviewing the literature, we found that the use of *ex vivo* uretero-pyeloscopy has been described for urolithiasis to remove stones before transplantation but not specifically to exclude malignancy. Ultimately, in this case, the patient underwent a renal autotransplantation for obstruction that was caused by a granuloma on the background of the previous BCG treatment. Intraoperatively, *ex vivo* uretero-pyeloscopy and frozen section were crucial in guiding this case by allowing for appropriate identification and resection of the ureteral lesion. In addition, the preservation of ureteral length allowed for autotransplantation, which remains effective at follow-up.

***Conclusion:*** *Ex vivo* urteroscopy has been used effectively in donor kidneys to treat urolithiasis with minimal complications. We believe that this is the first documented case of *ex vivo* uretero-pyeloscopy being used effectively in renal autotransplantation to exclude urothelial malignancy.

## Introduction and Background

Ureteroscopy and pyeloscopy (uretero-pyeloscopy) are common techniques that are used intraoperatively to view the ureter, as well as to the evaluation and treatment of benign conditions such as calculi and ureteral strictures. *Ex vivo* ureteroscopy (ExURS) involves examination of the ureter after removal of the kidney from the patient; most of the literature documents its use in the evaluation of donor kidneys and treatment of donor kidney stone disease.^1–4^

We utilized ExURS in the management of suspected malignancy to allow for autotransplantation and believe that this is a novel technique in modern urology that has not been previously documented. As such, a systematic review was performed to determine whether ExURS had previously been used in patients with suspected urothelial malignancy who were planned for autotransplantation.

We also describe our case where flexible ExURS was used on the back table of an autotransplantation to further assess and characterize a suspicious proximal ureteral lesion, allowing for effective resection of the lesion and use of the ureter for autotransplantation.

## Materials and Methods

**FIG. 1.**
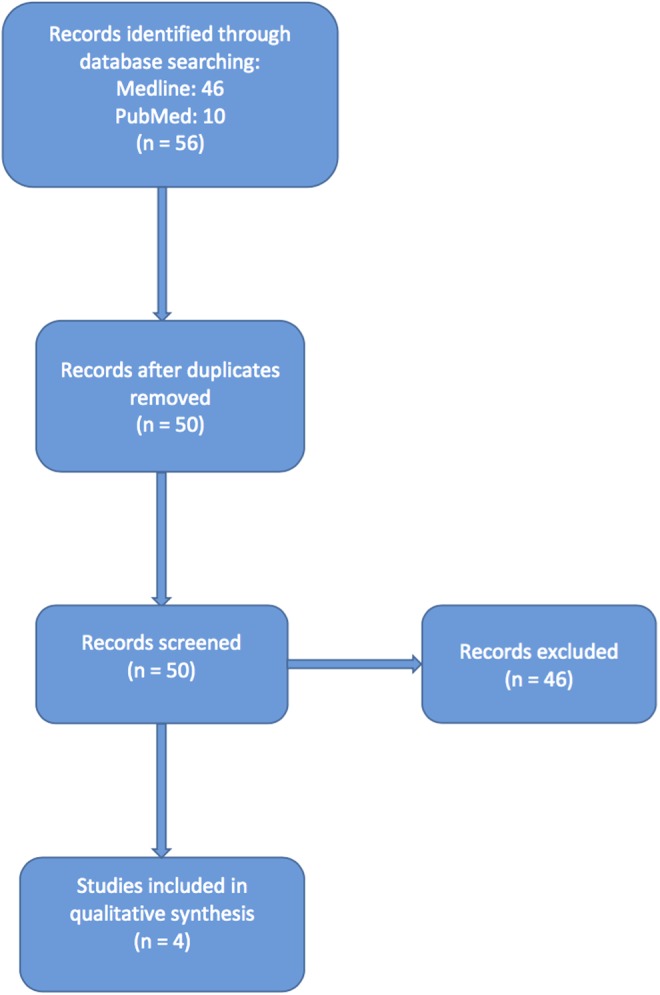
Flowchart of study selection as per the PRISMA statement.

A systematic review was undertaken based on guidelines as outlined by the Cochrane Collaboration and PRISMA statement (Fig. 1).

### Search strategy

A systematic literature search was performed using literature databases Medline through Ovid and PubMed, yielding the following results.

Medline 1946 to October week 1, 2015 using Ovid interface:

• [ex vivo ureteroscopy.mp], seven articles;
• [uretero pyeloscopy.mp], three articles;
• [ex vivo ureteroscopy.mp] and [autotransplantation], 0 articles.

PubMed

• [ex vivo ureteroscopy], 34 articles;
• [uretero pyeloscopy], 12 articles;
• [ex vivo ureteroscopy] and [autotransplantation], 0 articles.

A search of the literature was performed for the search term *ex vivo* ureteroscopy that yielded 46 articles on PubMed and 10 articles on Medline.

After review of the abstracts, duplicate articles were removed and those articles relating to the use of ExURS for training purposes on porcine models were also excluded. This resulted in four articles that discussed the role of ExURS in transplantation and treatment of donor kidney stone disease. Our search revealed no articles identifying the use of ExURS in autotransplantation or for the exclusion of urothelial malignancy.

## Presentation of Case

A 37-year-old female was found to have an incidental bladder lesion in 2007 at age 30 during ultrasonography investigation for subfertility. A transurethral resection of bladder tumor was performed, during which the lesion was found to extend into the proximal right ureter. Ureteroscopy was performed and the tumor was resected from the bladder and distal ureter. Histopathology showed a fibroepithelial polyp with no evidence of urothelial dysplasia or malignancy. Her medical history included two previous cesarean sections, lifelong nonsmoker, and no prior exposure to carcinogenic chemicals.

She subsequently developed recurrence of bladder tumors in 2010 and 2012 with the histopathology showing as papillary ureteritis with evidence of mitotic activity. She was commenced on a course of intravesical bacillus Calmette–Guerin (BCG) for the recurrence of her bladder tumors and the mitotic activity. Following completion of her BCG course, she remained tumor free at 3 and 6 months check with cystoscopies.

**FIG. 2.**
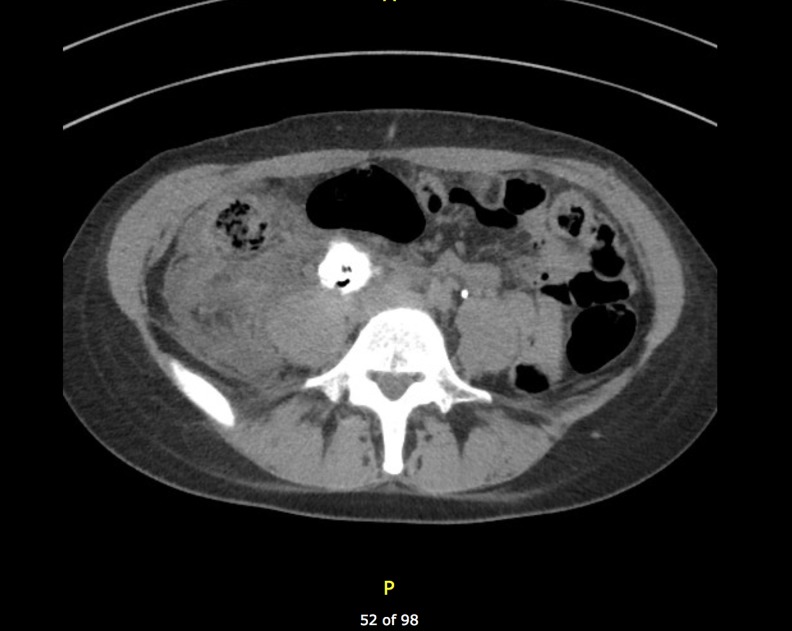
Preoperative CT showing right ureteral granuloma measuring 4.2 × 0.1 × 1.8 cm.

Eight months following BCG, she developed right-sided flank pain and although the bladder was clear of recurrence, a retrograde pyelogram was performed because of her previous distal right ureteral lesion. Although the distal ureter was clear, there was a new development of a large proximal ureter obstructing the polypoid lesion. A CT intravenous pyelogram showed that the lesion was 4.2 × 0.1 × 1.8 cm (Fig. 2). It is hypothesized that the previous resection at the right distal ureter had allowed reflux of the BCG proximally, stimulating the growth of this proximal ureteral granuloma.

Given the patient's age and history of recurrent bladder and ureteral tumors, a right nephroureterectomy and autotransplantation were carried out. A laparoscopic nephroureterectomy was performed that identified the right kidney with two renal arteries and a long renal vein with multiple segmental veins. The ureter was dissected away and the bladder stapled and the renal arteries and veins were ligated, with a warm ischemia time of 5 minutes.

**FIG. 3.**
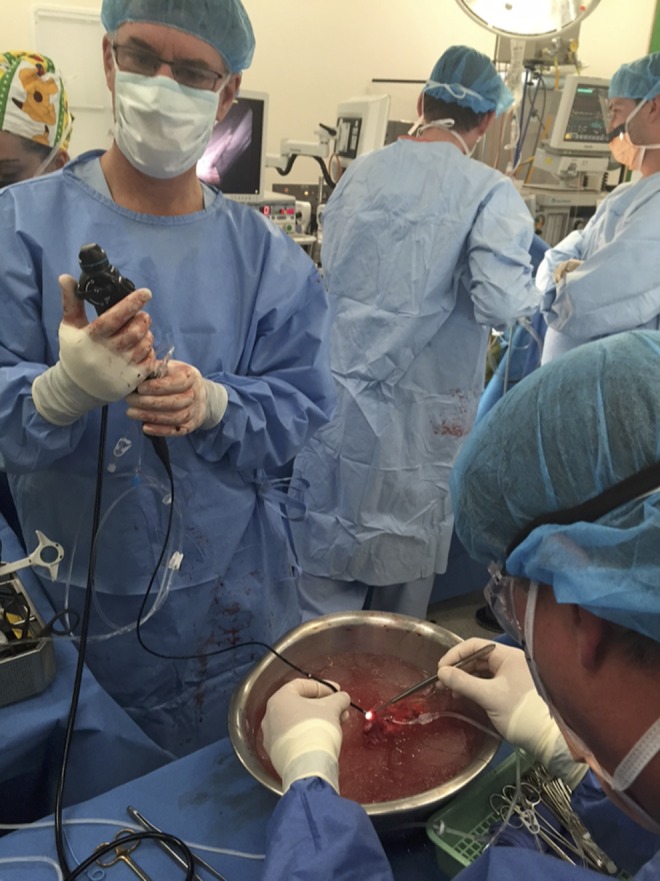
*Ex vivo* ureteroscopy being performed on a donor kidney by the authors (D.M.B. and N.L.) on back table before autotransplantation.

Following cold perfusion, back table exploration of the kidney was performed with flexible ExURS, which identified the proximal ureteral lesion (Fig. 3). ExURS confirmed the intraluminal lesion margins, allowing for resection of the lesion and distal ureter with adequate proximal length for reimplantation. Intraoperative frozen section of the proximal ureteral lesion shows inflamed granulation tissue with chronic histiocytic reaction, suggestive of a poorly formed granuloma.

Reimplantation of the kidney into the right pelvis was performed by a vascular surgeon, with anastomosis of the renal vein to the external iliac vein and the renal artery to the external iliac artery. The ureter was then reimplanted into the bladder by the urology team with 4-0 polydioxanone and a Double-J stent.

Postoperatively, the patient made an uncomplicated recovery with adequate urine output through an indwelling catheter. Her renal function on discharge was excellent with a serum creatinine of 58 μM and an estimated glomerular filtration rate of 91. She underwent an effective trial of void 2 weeks postoperatively and her stent was removed 3 weeks postoperatively. Formal histopathology of the ureteral tumor showed mucosal ulceration with moderate active chronic inflammation and granulomatous inflammatory reaction. Stains for acid-fast bacilli were negative and *Mycobacterium tuberculosis* complex polymerase chain reaction was also negative.

## Discussion

We believe that this is the first documented case where ExURS was used in a renal autotransplantation to exclude malignancy. In our case, ExURS was useful as it allowed back table inspection of the ureteral granuloma and its margins to guide our resection. This allowed adequate length of the ureter to be reimplanted into the bladder at the time of autotransplantation.

Other cases in the literature largely involve the use of ExURS in the treatment of stone disease in donor kidneys. Previously, the presence of calculi precluded the use of a kidney for transplantation, both cadaveric and living donor. Given the increasing number of those patients requiring transplantation and the shortage of donors, donor kidneys with calculi have been treated on the back table using ExURS.

In 2007, Trivedi et al. describe performing ExURS on two donor kidneys, one living and one cadaveric.^1^ Using ExURS, they were able to directly see the calculi and remove them using pneumatic fragmentation with forceps retrieval. In a retrospective review, Schade et al. performed ExURS on 23 kidneys from living donors, identifying calculi in 19 kidneys.^2^ They performed a combination of basket extraction and holmium laser lithotripsy under direct ExURS vision, with 17 kidneys being calculi-free after ExURS. The two cases that were unable to be calculi-free were related to infundibular stenosis of the calix and a narrow mouthed caliceal diverticulum. There were no intraoperative complications and they concluded that ExURS was safe and effective for calculi removal in live donor kidney allografts.

Although Schade et al. did not document any complications, Mosimann et al., in a letter to the editor in 2012, responded to Schade's study by describing a case of a 35-year-old patient receiving her father's right kidney.^3^ Following rigid ExURS to remove an 8-mm upper calix calculus through fragmentation and basket retrieval, the kidney was transplanted. However, initial revascularization was unsatisfactory and despite reanastomosis of the artery, the graft was explanted. The arterial anastomosis was widely patent, however, there was a major intimal flap in the hilum next to the renal pelvis—something that Mosimann attributed to the instrumentation of the renal pelvis, with a rigid ureteroscope causing damage to the neighboring artery.

Olsburgh et al. performed a retrospective analysis on incidental renal stones in live kidney donors and briefly discussed the use of ExURS. They identified 17 patients who underwent ExURS with basket retrieval or laser and identified no complications.^4^ Rashid et al. in 2004 examined 10 donor kidneys with calculi using ExURS and effectively treated them with endoscopic basket and fragmentation or laser lithotripsy, documenting no intraoperative or postoperative ureter-related complications.^5^

All of the previously listed articles mentioned the use of ExURS in being able to manipulate and rotate the kidney on the back table with a free ureter, allowing access to the calyces and calculi. They also used a combination of flexible and rigid ureteroscopes. In our case, a flexible ureteroscope was used to perform ExURS and this allowed manipulation of the kidney and ureter for further identification and characterization of the ureteral granuloma.

Although only a single case, our case demonstrates that ExURS can be used effectively to assess ureteral pathology in a kidney that is for autotransplantation. The limited literature on ExURS in the treatment of donor kidney calculi indicates that ExURS can be used to perform interventions on donor kidneys with minimal complication.

We conclude that ExURS is a useful technique in examining and treating donor kidney calculi with minimal documented complications. We utilized ExURS to examine a donor kidney that was for autotransplantation to exclude urothelial malignancy—a useful technique that has not previously been described.

## Abbreviations Used

BCG: bacillus Calmette–Guerin
ExURS: *ex vivo* ureteroscopy
